# Changes in cerebral metabolism in patients with a minimally conscious state responding to zolpidem

**DOI:** 10.3389/fnhum.2014.00917

**Published:** 2014-12-02

**Authors:** Camille Chatelle, Aurore Thibaut, Olivia Gosseries, Marie-Aurélie Bruno, Athena Demertzi, Claire Bernard, Roland Hustinx, Luaba Tshibanda, Mohamed A. Bahri, Steven Laureys

**Affiliations:** ^1^Coma Science Group, Cyclotron Research Centre, University and University Hospital of LiègeLiège, Belgium; ^2^Neurorehabilitation Lab, Department of Physical Medicine and Rehabilitation, Spaulding Rehabilitation Hospital, Harvard Medical SchoolBoston, MA, USA; ^3^Center for Sleep and Consciousness, and Postle Laboratory, Department of Psychiatry, University of WisconsinMadison, WI, USA; ^4^Nuclear Medicine Department, University Hospital of LiègeLiège, Belgium; ^5^Department of Neuroradiology, University Hospital of LiègeLiège, Belgium

**Keywords:** minimally conscious state, zolpidem, brain metabolism, positron emission tomography, prefrontal cortex, mesocircuit hypothesis

## Abstract

**Background:** Zolpidem, a short-acting non-benzodiazepine GABA agonist hypnotic, has been shown to induce paradoxical responses in some patients with disorders of consciousness (DOC), leading to recovery of arousal and cognitive abilities. We here assessed zolpidem-induced changes in regional brain metabolism in three patients with known zolpidem response in chronic post-anoxic minimally conscious state (MCS).

**Methods:** [18F]-fluorodeoxyglucose positron emission tomography (FDG-PET) and standardized clinical assessments using the Coma Recovery Scale-Revised were performed after administration of 10 mg zolpidem or placebo in a randomized double blind 2-day protocol. PET data preprocessing and comparison with a healthy age-matched control group were performed using statistical parametric mapping (SPM8).

**Results:** Behaviorally, all patients recovered functional communication after administration of zolpidem (i.e., emergence from the MCS). FDG-PET showed increased metabolism in dorsolateral prefrontal and mesiofrontal cortices after zolpidem but not after placebo administration.

**Conclusion:** Our data show a metabolic activation of prefrontal areas, corroborating the proposed mesocircuit hypothesis to explain the paradoxical effect of zolpidem observed in some patients with DOC. It also suggests the key role of the prefrontal cortices in the recovery of functional communication and object use in hypoxic patients with chronic MCS.

## Introduction

Following a severe brain injury, patients can stay in a prolonged period of unconsciousness. They can be in a coma for few days or weeks, before evolving into a vegetative/unresponsive wakefulness syndrome (i.e., eyes open but only showing reflex behaviors Laureys et al., 2010) or into a minimally conscious state (MCS, Giacino et al., 2002). MCS is characterized by inconsistent but reproducible evidence of awareness (e.g., signs of consciousness without command following- MCS minus; with command following- MCS plus, Bruno et al., 2011). Emergence from MCS (EMCS) is characterized by the recovery of functional communication and/or functional object use. According to the Coma Recovery Scale-Revised assessment (CRS-R, Giacino et al., 2004), functional communication is reached when a patient is able to answer 6 out of 6 “yes–no” questions accurately. Communication is intentional if (s)he is able to answer at least 2 out of 6 questions independently of accuracy. Functional object use encompasses the ability to accurately use two different objects two times. Research on efficient treatments improving cognitive abilities in this population of patients with MCS has shown that deep brain stimulation (Schiff et al., 2007) and some pharmacological agents such as amantadine (Schnakers et al., 2008a; Giacino et al., 2012), apomorphine (Fridman et al., 2009) intrathecal baclofen (Sara et al., 2007) and zolpidem (Whyte and Myers, 2009; Thonnard et al., 2013) can improve recovery in some cases (for a recent review, see Gosseries et al., 2013). However, the mechanisms underlying these recoveries remain poorly understood.

Zolpidem is a short-acting non-benzodiazepine agent from the imidazopyridine class usually used against insomnia (Langtry and Benfield, 1990; Sanger, 2004). It has been shown to induce paradoxical responses in some patients with disorders of consciousness (DOC), leading to an improvement of arousal and cognitive abilities. Several case-studies showed that it can induce very impressive recoveries in severely brain-damaged patients with DOC of various etiologies (Clauss et al., 2000; Cohen et al., 2004; Clauss and Nel, 2006; Brefel-Courbon et al., 2007; Shames and Ring, 2008; Williams et al., 2013). However, this effect remains rare (i.e., around 5–7% of responders; Whyte and Myers, 2009; Thonnard et al., 2013; Whyte et al., 2014) and it is not always significant in terms of functional recovery (in the 7% of the patients showing improvement, no diagnosis changes was observed in Thonnard et al., 2013). A change in brain activity (e.g., prefrontal cortices, thalami and striatum) after zolpidem intake has been reported in single subject studies using single-photon emission computed tomography (measuring blood flow) (Clauss et al., 2000), positron emission tomography (PET, Brefel-Courbon et al., 2007; Williams et al., 2013; measuring blood flow and metabolism), and electroencephalography (EEG; measuring electrical activity Williams et al., 2013).

**Figure 1:**
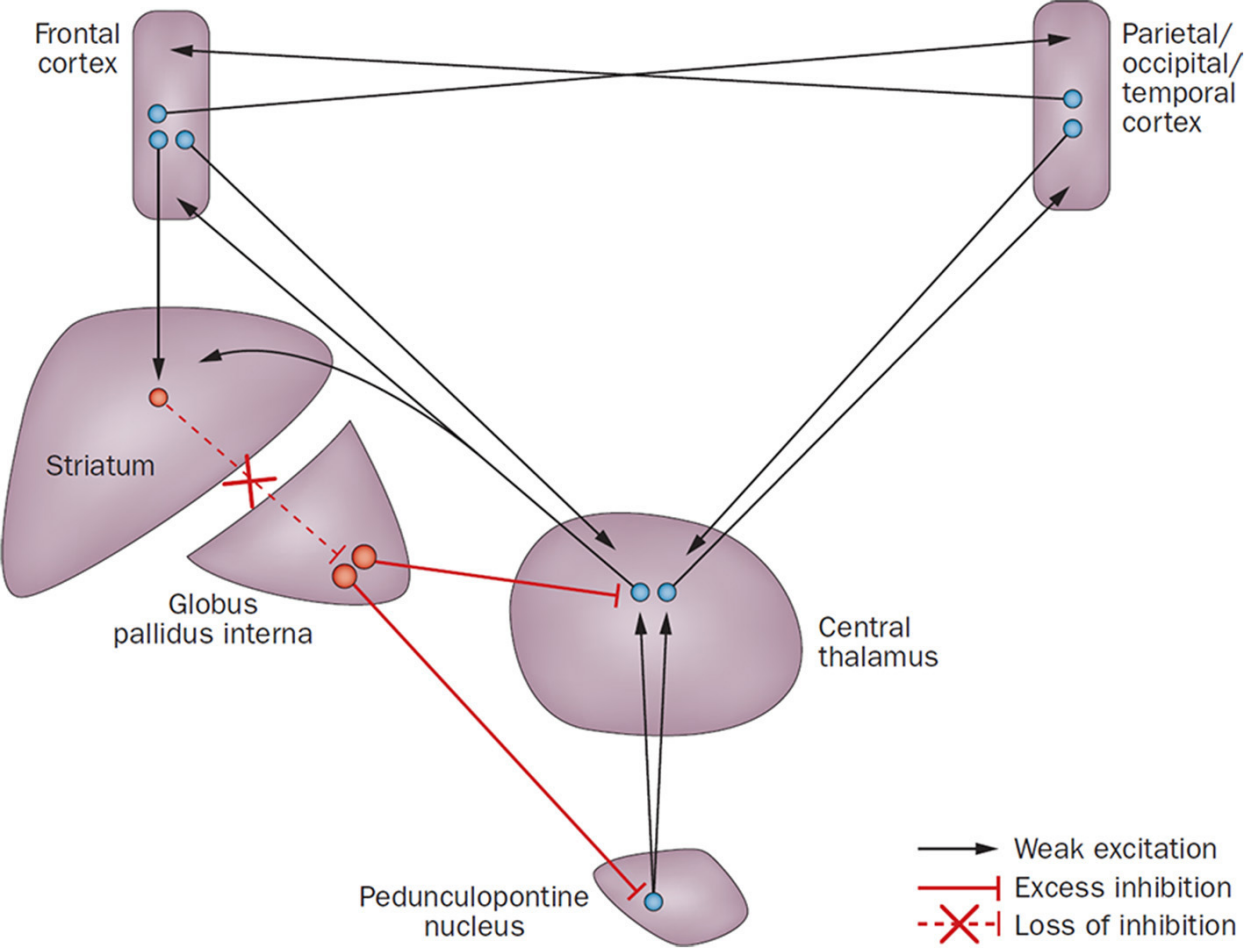
**The mesocircuit model underlying forebrain dysfunction and interventions in severe brain injuries (From Giacino et al., 2014, with permission)**. Reduction of thalamocortical and thalamostriatal outflow following deafferentation and neuronal loss from the central thalamus withdraws the afferent drive to the striatum, which may then fail to reach firing threshold because of their requirement for high levels of synaptic background activity. Loss of active inhibition from the striatum allows neurons of the globus pallidus interna to tonically fire and provide active inhibition to their synaptic targets, including relay neurons of the already disfacilitated central thalamus, and possibly also the projection neurons of the pedunculopontine nucleus. Since the GABAA a-1 subunit is normally expressed in large quantities in the globus pallidus interna, zolpidem could inhibit the latter, substituting its normal inhibition from the striatum, and hence induce an increase of the thalamic excitatory influence on prefrontal cortices.

We here assessed zolpidem-induced changes in regional brain metabolism in a case-series of three patients with known zolpidem response after chronic post-anoxic MCS. According to the mesocircuit model for the recovery of consciousness (Schiff, 2010), zolpidem is suggested to disinhibit the globus pallidus interna (GPi) and by that way increase the thalamic excitatory role on prefrontal cortices (see Figure 1). Based on this model, we hypothesized that an impaired brain metabolism in the thalamus, striatum and prefrontal areas would be observed at the group level during placebo, which would recover following zolpidem intake.

## Materials and methods

Three patients with chronic post-anoxic MCS with known clinical improvement to zolpidem administration (i.e., EMCS) were included in the study. All patients took zolpidem on a regular basis for at least 6 months. For the present study, zolpidem intake was stopped at least 12 h prior to the research protocol. 10 mg of zolpidem or placebo (water) were administered at 12 a.m. via gastrostomy in a randomized order, in a double blind 2-day design. All other treatments remained unchanged throughout the study. Standardized clinical assessment using the CRS-R (Giacino et al., 2004; Schnakers et al., 2008b) was performed 30 min after administration of zolpidem or placebo. The CRS-R is a validated and sensitive behavioral assessment scale to determine patients' level of consciousness (Seel et al., 2010). It assesses auditory, visual, verbal, and motor functions as well as communication and arousal level. The total score ranges between 0 (coma) and 23 (EMCS).

FDG-PET cerebral metabolism data were acquired 90 min after zolpidem or placebo intake, at the University Hospital of Liège using a Gemini TF Big Bore (Philips Medical System) and according to a standard clinical protocol. An intravenous injection of 300 MBq fluorodeoxyglucose was administered 30 min before the FDG-PET. Patients were monitored by an anesthesiologist throughout the procedure. The study was approved by the Ethics Committee of the Faculty of Medicine of the University of Liège and written informed consent was obtained from the patients' legal representatives and all volunteers.

PET data of patients were compared to an age-matched group of 39 healthy participants (mean age 45 ± 16 years; 18 men). Preprocessing of the PET data was identical as previously published (Phillips et al., 2011; Bruno et al., 2012; Thibaut et al., 2012) including spatial normalization and smoothing (using a 14 mm full width at a half maximum Gaussian kernel), implemented in Statistical Parametric Mapping toolbox (SPM8; www.fil.ion.ucl.ac.uk/spm).

The statistical analyses were also performed using SPM8 toolbox. A full-factorial design with three design matrices modeled the subject-effect (MCS patient 1, 2, 3), drug-effect (placebo vs. zolpidem) and group effect (patients vs. controls) was performed. After proportional scaling, we identified brain regions that showed a relative increase in metabolism after zolpidem intake as compared to placebo. Areas showing an impaired metabolism after placebo and after zolpidem intake as compared to healthy controls were also investigated. Results were considered significant at false discovery rate cluster level *p* < 0.001 after a Bonferroni correction for multiple comparisons.

### Case reports

Table 1 shows the CRS-R subscores after placebo and zolpidem intake.

**Table 1:**
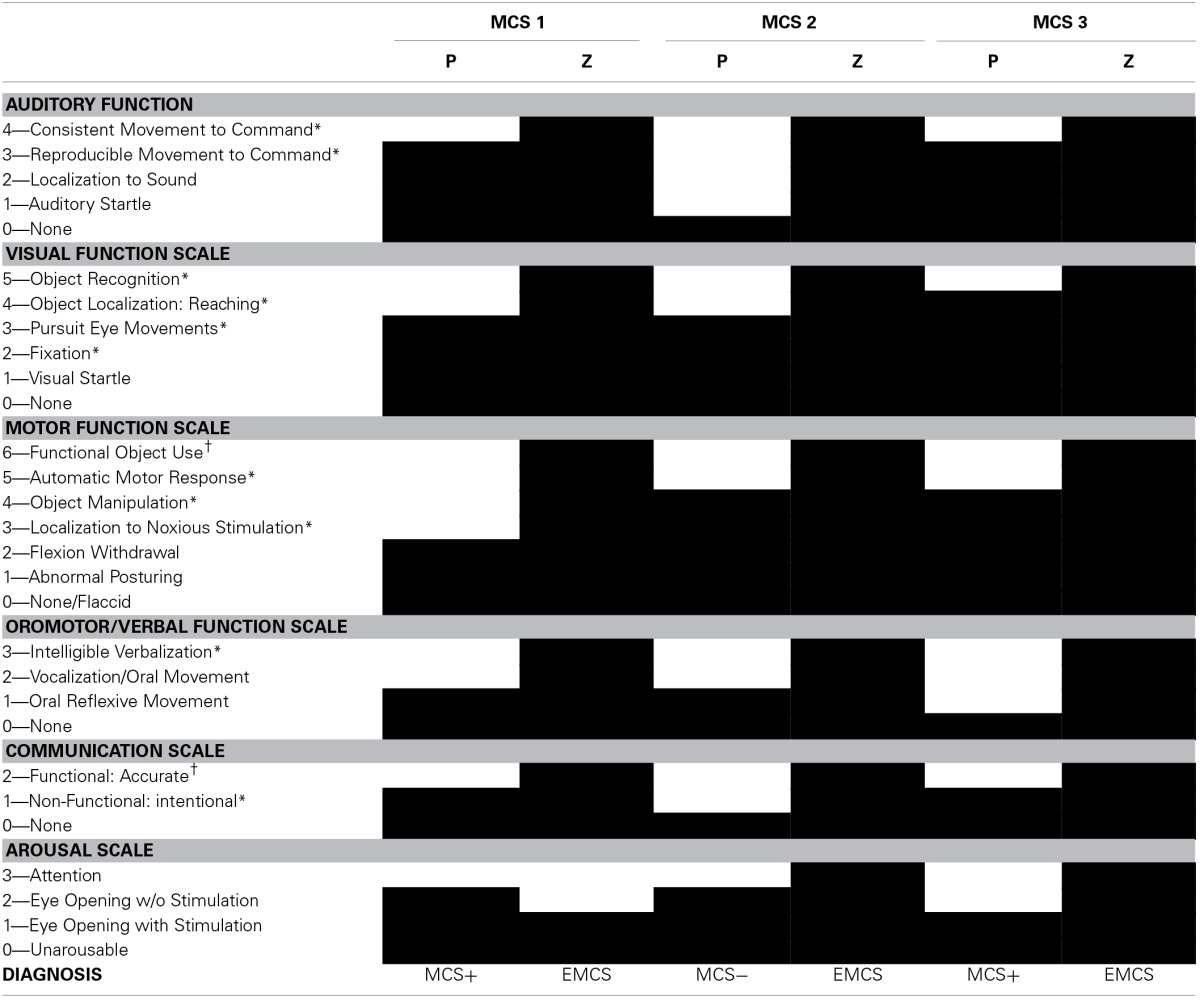
**Behavioral assessments after placebo and zolpidem intake (based on the Coma Recovery Scale-Revised)**.

MCS−, minimally conscious state minus (i.e., non reflex movements without response to command); MCS+, minimally conscious state plus (i.e., presence of response to command); EMCS, emergence from the minimally conscious state (i.e., functional communication or object use).

^*^Indicates clinical signs compatible with MCS. ^†^Indicates emergence from minimally conscious state. P, placebo; Z, zolpidem. Highest score achieved for each subscale is represented by the black boxes.

### MCS 1

A 37-year-old female was assessed 18 months post anoxia (cardio-respiratory arrest after hanging). Within the month post-insult, she emerged from the vegetative state/unresponsive wakefulness syndrome and evolved into a MCS plus. CRS-R assessment showed reproducible but not consistent command following, visual pursuit, and intentional communication. MRI showed ischemic brain lesions in the basal ganglia, in the left occipital and bilateral posterior parietal cortices. Resting EEG showed a reactive background rhythm of alpha/theta activity (7–8 Hz) without any epileptic activity. After zolpidem administration, she could systematically follow simple commands, recognize different objects and use them adequately, and communicate functionally (i.e., EMCS). Concurrent treatment consisted of amantadine, fluoxetine, trihexyphenidyl, tramadol, and esomeprazole.

### MCS 2

A 38-year-old male was assessed 12 years and 7 months post-anoxia (cardio-respiratory arrest after hanging). The patient showed visual pursuit and automatic motor reactions (i.e., mouth opening when a spoon was brought to his mouth), and was diagnosed as MCS minus. The MRI showed lesions in the brainstem and in the thalami with diffuse periventricular white matter damage, more pronounced in posterior regions and in the left hemisphere. Resting EEG showed a background rhythm of 7 Hz theta activity without epileptic activity. After zolpidem administration, he was able to systematically follow simple commands, recognize objects and use them consistently, and communicate functionally (i.e., EMCS). Concurrent treatment consisted of levetiracetam.

### MCS 3

A 50-year-old female was assessed 7 years post anoxia (cardio-respiratory arrest after hanging). Structural MRI did not show focal abnormalities (PET glucose and activation blood-flow data have been reported elsewhere, Brefel-Courbon et al., 2007). She remained 3 weeks in a coma before recovering signs of consciousness. CRS-R assessment showed automatic motor reactions (i.e., pulling on her shirt), reproducible but not consistent command following, localization of objects, and intentional communication (i.e., MCS plus). Resting EEG recordings did not demonstrate any epileptic discharges with a background rhythm of 6 Hz theta activity. Following zolpidem intake, she showed consistent command following, functional use of objects, and functional communication (i.e., EMCS). Concurrent treatment consisted of modafinil, fluoxetine, piribedil, dihydroergocristine, domperidone, vinburnine, heptaminol, and omeprazole.

## Results

**Figure 2:**
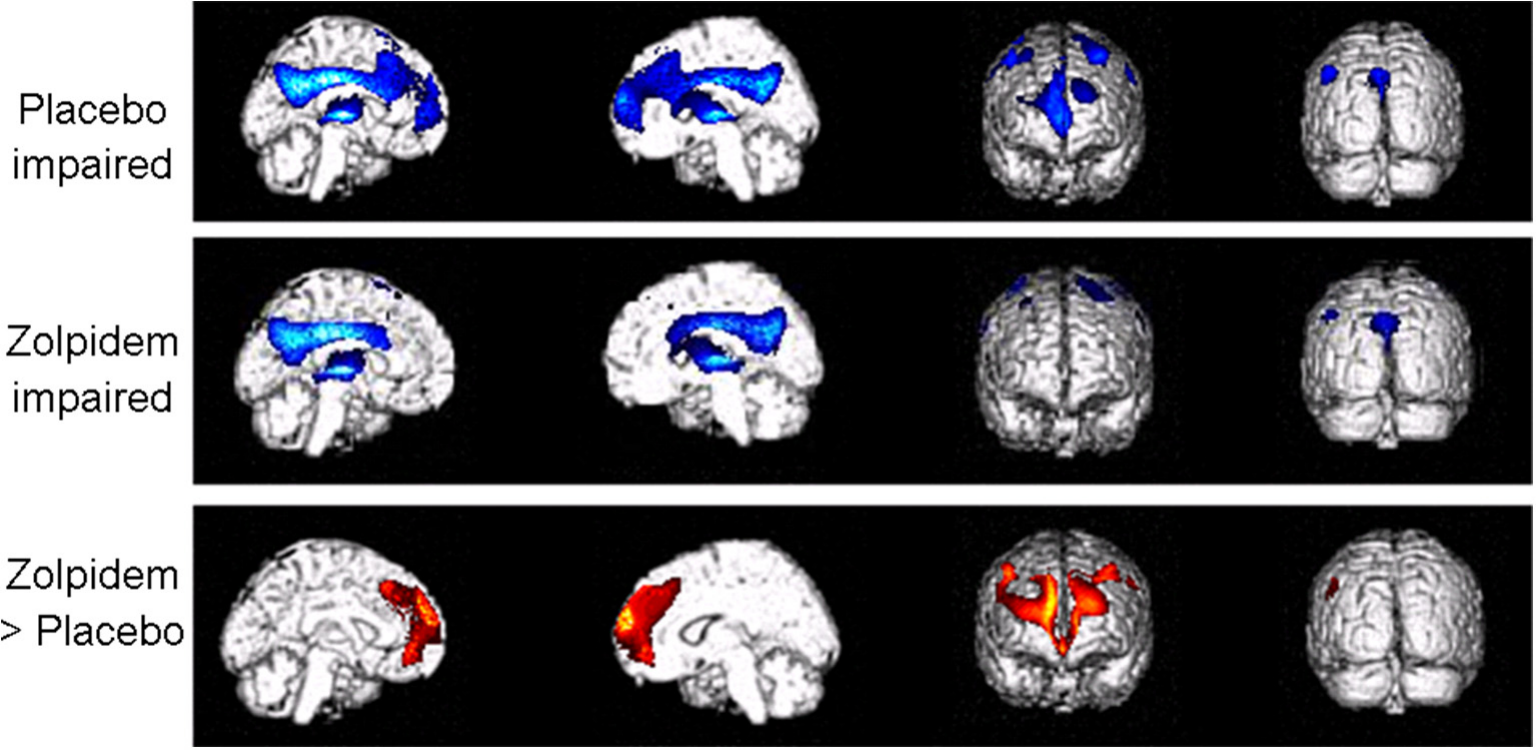
**Impaired brain metabolism after placebo and zolpidem intake and areas showing relative recovery after zolpidem**. Brain areas showing impaired metabolism (in blue) following placebo and zolpidem administration and regions which were impaired following placebo but showed relative recovery of activity after zolpidem intake (in red). For display purposes results are shown thresholded at uncorrected *p* < 0.001. From left to right, medial right and left view, frontal and posterior view of a 3D rendered brain MRI.

**Figure 3:**
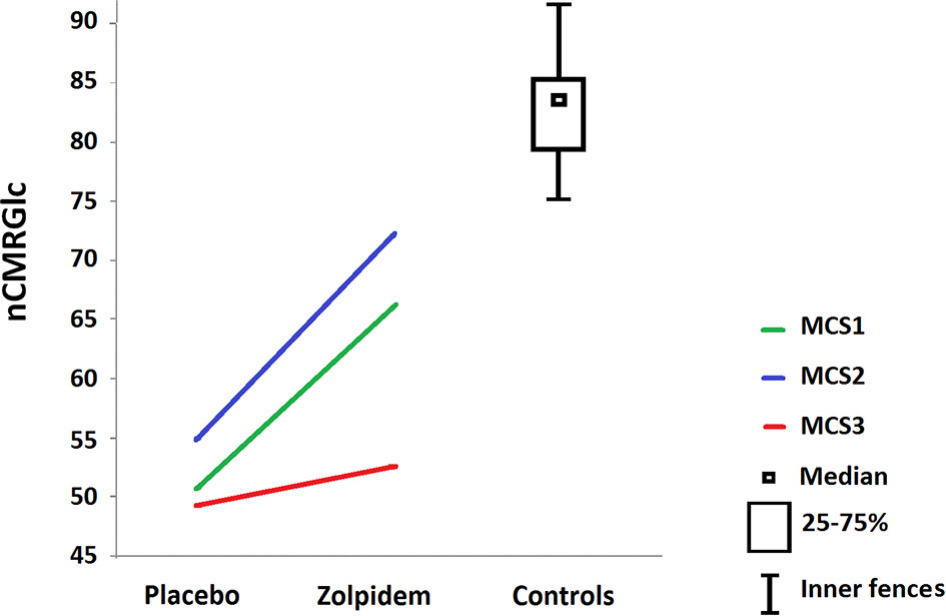
**Normalized cerebral metabolic rate for glucose (nCMRGlc) values in prefrontal cortices after placebo**. Values reported for the cluster using peak coordinates *x*, *y*, *z* = −22, 56, 24 after placebo and zolpidem intake in patients, as compared to healthy controls (boxplot showing median, 25–75% quartiles and inner fences).

Group level analysis showed a relative increase in the bilateral superior frontal gyri and the right medial frontal cortex following zolpidem intake as compared to placebo (Table 2 and Figures 2, 3). At a less conservative statistical threshold uncorrected for multiple comparisons, the left insula, the middle frontal gyri and the left inferior frontal and parietal areas also showed a relative increase following zolpidem intake as compared with placebo. During placebo, the thalami and the left precuneus/posterior cingulate areas showed an impaired brain metabolism as compared with healthy controls. At a less conservative statistical threshold, bilateral superior and middle frontal gyri, the left precuneus/posterior cingulate, bilateral precentral gyri, left insula and right inferior parietal areas showed an impaired brain metabolism. After zolpidem intake, the thalami and the left precuneus/posterior cingulate areas showed an impaired brain metabolism as compared with healthy controls. At a less conservative threshold, the precentral gyri, left superior frontal and temporal gyri, the left middle frontal gyrus and precuneus and the right inferior parietal lobe showed an impaired brain metabolism.

**Table 2:** **Regions showing significant impaired metabolism following placebo and zolpidem and relative increase following zolpidem intake**.

**Region (Brodmann area)**	***X* (mm)**	***Y* (mm)**	***Z* (mm)**	***Z*-value**	***P*-value**
**IMPAIRED AFTER PLACEBO**					
Right thalamus	12	−16	10	6.15	≤0.0001^**^
Left thalamus	−12	−16	12	6.11	≤0.0001^**^
Left posterior cingulate cortex (B31)	−2	−28	34	5.98	≤0.0001^**^
Left middle frontal gyrus (B6)	−34	18	60	4.00	≤0.0001^*^
Left superior frontal gyrus (B6/10)	−20	14	70	3.64	≤0.0001^*^
Left precuneus (B19)	−44	−70	42	3.93	≤0.0001^*^
Right precentral gyrus (B6)	36	−12	70	3.86	≤0.0001^*^
Right middle frontal gyrus (B6)	48	12	56	3.63	≤0.0001^*^
Right superior frontal gyrus (B8)	32	30	56	3.50	≤0.0001^*^
Left insula (B13)	−46	6	−2	3.51	≤0.0001^*^
Right inferior parietal lobe (B40)	64	−32	48	3.25	≤0.001^*^
Left precentral gyrus (B6)	−38	−16	68	3.19	≤0.001^*^
**IMPAIRED AFTER ZOLPIDEM**					
Right thalamus	12	−16	10	6.34	≤0.0001^**^
Left thalamus	−10	−16	10	6.24	≤0.0001^**^
Left posterior cingulate cortex (B31)	−2	−28	34	5.95	≤0.0001^**^
Right precentral gyrus (B6/4)	36	−12	70	4.02	≤0.0001^*^
Left superior frontal gyrus (B6/8)	−14	16	70	9.77	≤0.0001^*^
Left middle frontal gyrus (B6)	−32	20	60	3.63	≤0.0001^*^
Left precuneus (B19)	−42	−70	44	3.57	≤0.0001^*^
Left precentral gyrus (B6)	−38	−16	68	3.51	≤0.0001^*^
Right inferior parietal lobe (B40)	64	−30	48	3.37	≤0.0001^*^
Left superior temporal gyrus (B22)	−46	4	−4	3.29	≤0.0001^*^
**RELATIVE INCREASE AFTER ZOLPIDEM AS COMPARED TO PLACEBO**					
Left superior frontal gyrus (B10)	−22	56	24	4.35	≤0.0001^**^
Right superior frontal gyrus (B10)	12	60	20	4.35	≤0.0001^**^
Right medial frontal gyrus (B9)	8	42	32	4.05	≤0.0001^**^
Left insula (B13)	−44	14	2	3.37	≤0.0001^*^
Left inferior frontal gyrus (B47)	−34	20	−8	3.32	≤0.0001^*^
Left middle frontal gyrus (B8)	−54	14	42	3.34	≤0.0001^*^
Left angular gyrus (B39)	−46	−62	34	3.22	≤0.001^*^
Right middle frontal gyrus (B9)	44	18	28	3.24	≤0.001^*^
Left inferior parietal lobe (B40)	−44	−54	42	3.22	≤0.001^*^

coordinates of peak voxels in standardized stereotaxic MNI space.

** Corrected p-value for multiple comparisons.

* Uncorrected p-value.

## Discussion

Using a double-blind placebo-controlled design, we here report FDG-PET changes after the administration of zolpidem in three chronic post-anoxic patients in MCS who show clinically significant paradoxical behavioral improvements (i.e., EMCS). We observed that zolpidem-related recovery of cognitive abilities was paralleled by an increase in glucose metabolism in bilateral dorsolateral prefrontal and mesiofrontal cortices. Our findings support previous case-studies reporting a change in prefrontal cortex activity after zolpidem intake using single-photon emission computed tomography (Clauss et al., 2000), PET (Brefel-Courbon et al., 2007; Williams et al., 2013) and EEG (Williams et al., 2013). Prefrontal areas are known to be involved in the “limbic loop” regulation of motivation and are a key center of the mesocircuit model for consciousness (Schiff, 2010; Laureys and Schiff, 2012). The mesocircuit hypothesis for the effect of zolpidem (Schiff, 2010) supports the idea that, in normal cognitive processing, the striatum disinhibits the central thalamus via the internal globus pallidus (GPi) while the central thalamus promotes activity of prefrontal cortices. Therefore, if the activity in the striatum is reduced (e.g., following a severe brain injury), central thalamic and prefrontal activities are also reduced, possibly explaining the observed hypometabolism of the latter in the here reported patients in the baseline condition (i.e., after placebo administration). Since the GABAA a-1 subunit is normally expressed in large quantities in the GPi, zolpidem could inhibit the GPi, substituting the normal inhibition of the GPi from the striatum. The mesocircuit model assumes that zolpidem directly inhibits the GPi, substituting the normal inhibition of the GPi from the striatum (one of the most sensitive areas to cerebral hypoxia Calabresi et al., 2000) and would hence increase the thalamic excitatory influence on prefrontal cortices. Interestingly, in all studies with zolpidem responders (Clauss et al., 2000; Brefel-Courbon et al., 2007), including ours, the brain areas showing increased metabolism after zolpidem did not show significant structural lesions. This could support the idea that patients who respond to zolpidem have neurologic deficits mainly caused by inhibitory functional effects rather than by severely structurally damaged or dead brain tissue (Shames and Ring, 2008). This effect is defined as cerebral *diashisis*, i.e., the loss of function in a portion of the brain (here, the prefrontal cortices) as a result of its connection to another injured area (here, striatum and/or thalami) (Glassman, 1971; Feeney and Baron, 1986; Tecco et al., 1998; Witte et al., 2000). It has been proposed that zolpidem may be effective to restore brain function in patients whose brain injuries are mainly located outside of brainstem structures (Du et al., 2014). However, in our three responders one did show structural brainstem lesions (Du et al., 2013). In contrast to two previous case-studies (Brefel-Courbon et al., 2007; Williams et al., 2013), we did not observe a zolpidem-related increase in metabolism in thalamic and striatal regions. This could be explained either by the fact that the technique used is not sensitive enough to highlight small functional activity changes in these areas, or because zolpidem induced changes might modify effective connectivity between areas which cannot be investigated using FDG-PET. Future neuroimaging studies looking at effective connectivity in DOC zolpidem-responders would allow to better document the role of thalamo-cortico excitatory pathways underlying the observed increased activity in prefrontal areas. In addition, further studies focusing on regional glucose metabolism changes at the individual level in specific areas involved in the mesocircuit would help to better understand the mechanisms of recovery following zolpidem intake (Fridman et al., 2014).

Another theory, the “GABA impairment hypothesis” was recently proposed by Pistoia et al. (2014) to explain the effect of zolpidem on recovery of consciousness. This theory suggests that zolpidem (as well as baclofen, a GABA-B agonist) may act on consciousness recovery thanks to the restoration of a normal ratio between synaptic excitation and inhibition by reversing the impairment of GABA in this population of severely brain-injured patients. This hypothesis has the advantage to explain the potential mode of action of both zolpidem and baclofen on patient's recovery. In some patients with an impaired balance of cortical subcortical-cortical connections, the use of GABA agonists would decrease excessive information and thus reorganize an equilibrate dialogue among different brain nuclei and allow proper information processing. Nevertheless, the time course of the observed effect of these two drugs is different. For zolpidem, the short-term effect that disappear when the plasma drug concentration falls suggest rapid neurotransmitter changes. On the other hand, for baclofen, its slow-onset effects suggest a phenomenon of gradual neuroplasticity. Unfortunately, we cannot verify this hypothesis with the method used in our study (i.e., FDG-PET) this will therefore need to be addressed in future studies.

Finally, if all these theories can explain part of the mechanisms underlying the paradoxical responses induced by zolpidem, they do not explain why only less than 10% of the patients react positively to those GABA agonist drugs. Large cohort studies may help investigating this question.

We have to highlight that our study included only three patients, and all had a hypoxia; therefore, our results are not generalizable to the general DOC population (Adams et al., 2000). Note also that not all the patients with post-anoxic DOC may improve after zolpidem. In addition, the three patients were very likely to suffer from depression prior to injury (as they all attempted suicide), which may be a confounding factor. One could argue that vigilance in MCS patients is fluctuating and the behavioral improvement observed in the three patients could be related to this bias. Nevertheless, these patients were in a chronic stage (i.e., more stable in their responses) and they showed a dramatic recovery of functional communication/object use that has not been observed before. These elements support the idea of a diagnostic change induced by zolpidem intake.

Our results also underline a key role of the prefrontal cortices in the recovery of functional communication and object use in those hypoxic patients with chronic DOC. It is well known that the prefrontal cortex has a major role in executive function and working memory, a recovery of its functionality is therefore likely to influence object use and recovery of communication. Our findings partly corroborate previous case-studies (Nakayama et al., 2006; Thibaut et al., 2012) reporting a decreased metabolism in medial prefrontal and frontobasal regions, cingulate gyrus and thalamus as a function of the consciousness impairments in severely brain-injured patients. In addition, we observed a relative decrease in brain metabolism in the left superior temporal gyrus following zolpidem intake. This could be explained by the reestablishment of top-down processes associated with the reactivation of the prefrontal areas following zolpidem.

Taken together, the data suggest that the infrequent but existing paradoxical effect of zolpidem could be characteristic to patients having suffered subcortical thalamic (as in all 3 cases here reported) and/or striatal functional lesions preventing prefrontal cortices to exhibit their normal function.

## Conclusion

We observed an increased metabolism in prefrontal cortices following zolpidem intake, in line with the previously proposed mesocircuit model for recovery of consciousness in DOC (Schiff, 2010). Large cohort studies comparing brain structure, function and mesocircuit connectivity of DOC zolpidem-responders as compared to the vast majority of non-responders would allow to better understand in which cases zolpidem might improve cognitive function in such a clinically significant and yet transient manner. Increasing our research efforts to understand the common denominator and neural mechanisms underlying these rare cases responding to zolpidem could provide new avenues for promoting therapeutic strategies improving the recovery of some DOC patients with severe acquired brain damage.

## Author contributions

Camille Chatelle designed the study, performed the statistical analysis and drafted the manuscript with Aurore Thibaut. Olivia Gosseries participated in medical data collection and helped drafting the manuscript. Mohamed A. Bahri participated in the design of the study and acquired behavioral and PET data. Athena Demertzi collected behavioral and MRI data. Claire Bernard and Roland Hustinx performed PET examination and preprocessed the data. Luaba Tshibanda did the visual analysis of structural MRI data. Marie-Aurélie Bruno revised the manuscript critically for important methodological content. Steven Laureys participated in the design of the study and revised the manuscript critically for important intellectual content. All authors read, commented and approved the final manuscript.

### Conflict of interest statement

The authors declare that the research was conducted in the absence of any commercial or financial relationships that could be construed as a potential conflict of interest.
